# Artificial Termite-Fishing Tasks as Enrichment for Sanctuary-Housed Chimpanzees: Behavioral Effects and Impact on Welfare

**DOI:** 10.3390/ani11102941

**Published:** 2021-10-11

**Authors:** Maria Padrell, Federica Amici, Maria Pau Córdoba, Albert Giberga, Antonio Broekman, Susana Almagro, Miquel Llorente

**Affiliations:** 1 Departament de Psicologia, Facultat d’Educació i Psicologia, Universitat de Girona, 17004 Girona, Spain; miquel.llorente@gmail.com; 2 Unitat de Recerca i Etologia, Fundació Mona, 17457 Riudellots de la Selva, Spain; mp.cordoba.a@gmail.com; 3 Department of Human Behavior, Max Planck Institute for Evolutionary Anthropology, Ecology and Culture, Deutscher Platz 6, D-04103 Leipzig, Germany; amici@eva.mpg.de; 4 Faculty of Life Science, Institute of Biology, University of Leipzig, Talstrasse 33, D-04103 Leipzig, Germany; 5 Fundació UdG, Innovació i Formació, Universitat de Girona, Carrer Pic de Peguera 11, 17003 Girona, Spain; gibergaalbert@gmail.com (A.G.); antobroekman@hotmail.com (A.B.); susanalmagro@gmail.com (S.A.)

**Keywords:** chimpanzees, behavior, enrichment, tool use, welfare

## Abstract

**Simple Summary:**

The welfare of captive animals is nowadays a topic of major concern. In order to express their natural behavioral repertoires, however, animals require complex environments and stimuli which are difficult to reproduce in captivity. To overcome this, environmental enrichment is considered one of the most successful tools to increase behavioral opportunities and enhance animal welfare. In this study, we explored whether providing an artificial termite-fishing task, and whether participation in this task, predicted changes in the solitary and social behavior of sanctuary-housed chimpanzees (*Pan troglodytes*). We compared chimpanzee behavior when the enrichment was presented to different periods without enrichment. We found that the presence of the enrichment predicted an increase in tool use and feeding behavior and a decrease in inactivity, especially for those chimpanzees with higher participation. However, we did not detect significant changes in abnormal or self-directed behaviors. Furthermore, we found no variation in affiliation- or aggression-related behaviors, but social proximity increased in chimpanzees that participated more. Our results support previous studies demonstrating that artificial termite-fishing promotes species-typical behaviors in captive chimpanzees with no major effects on social activities.

**Abstract:**

Artificial termite-fishing tasks are a common enrichment for captive great apes, promoting species-typical behaviors. Nonetheless, whether these activities are linked to changes in other behaviors and whether these changes persist over time has seldom been investigated. We assessed whether the use of an artificial termite-fishing task was linked to changes in the solitary behavior and social dynamics in two groups of sanctuary-housed chimpanzees (*Pan troglodytes*). Specifically, we compared chimpanzee behavior during eight enrichment sessions distributed over a two-month period, with similar periods before and after the introduction of the enrichment. Data were collected from combined interval and continuous sampling methods and were analyzed using generalized linear mixed models. We found that participation increased across sessions and that both enrichment and participation predicted an increase in tool use and feeding and a decrease in inactivity, which were all maintained throughout the sessions. Furthermore, participation was positively associated with social proximity, revealing a gathering effect of the task. However, neither enrichment nor participation were linked to changes in abnormal, self-directed, affiliation-related or aggression-related behaviors. Overall, our results support the hypothesis that artificial termite-fishing is a suitable enrichment for captive chimpanzees, maintaining the subjects’ interest and promoting species-typical behaviors, with no negative effects on social activities.

## 1. Introduction

Concern for the welfare of captive animals has progressively increased over the past few decades. Although early studies mainly focused on farm animals [1,2], the welfare of zoo and sanctuary animals has, more recently, become a topic of major interest, as shown by the increasing number of scientific articles on the topic [3,4,5,6,7,8], as well as the development of specific guidelines and recommendations on how to assess and improve animal welfare [9]. Similarly, there has been a rise in awareness among zoological institutions, so that welfare is now considered a key factor in animal management [10].

Welfare has been conceptualized as the state of an individual in relation to its attempts to cope with its environment [11]. However, in order to achieve an optimal level of welfare, animals not only need to cope with their environment but also thrive in it [8,12]. In other words, they need to be provided with opportunities to experience positive welfare states to have a “life worth living” [13]. In particular, they must be able to express species-typical behaviors, also known as ethological needs [14,15], or natural behaviors [16] like they exhibit in the wild [17]. Nonetheless, adequate conditions for the expression of a species natural behavioral repertoire demand complex environments and stimuli [18,19,20]; unfortunately, captive settings often fail to fulfill these requirements. To overcome this, environmental enrichment is generally considered one of the most effective tools to increase behavioral opportunities and enhance welfare; thus, they are a key component of captive animal management [12]. The main goals of environmental enrichment include increasing behavioral diversity, promoting natural behavioral patterns and reducing the occurrence of abnormal behaviors [18]. Furthermore, environmental enrichment may increase positive affective states [21], generate highly motivated behaviors and modify the physiological response of animals [22].

In order to assess the impact of a particular enrichment strategy, researchers usually monitor their subjects’ behavior by comparing the frequency of species-typical behaviors and abnormal/stress-related behaviors in the presence (versus in the absence) of the enrichment [16,23]. To date, a wide range of environmental enrichment strategies has been implemented and evaluated in non-human primates, including enclosure design and size, food novelty, foraging devices, computer-based devices, sensory stimulation or exposure to conspecifics or humans (i.e., social enrichment) [24,25]. Because wild non-human primates usually spend more time foraging or feeding than captive primates, it is not surprising that enrichment activities for captive groups have mainly focused on increasing opportunities for foraging or feeding [26,27,28]. Extractive foraging devices requiring tool use, for instance, are popular enrichments among captive great apes, especially in chimpanzees [28,29,30,31,32,33]. In great apes, foraging devices often simulate ant- or termite-fishing, as observed in the wild [34,35]. These activities have been mainly used to study cognitive aspects of tool use, such as acquisition and learning [36,37,38], tool modification [39], flexibility [40] or laterality [41]. However, several authors have also assessed the impact of these enrichment activities on chimpanzee welfare. As expected, these studies found an increase in the frequency of tool use in the presence of the enrichment, as well as changes in other behaviors. For example, Celli and colleagues [29] found that besides predicting an increase in chimpanzee manipulation and tool use, ant-fishing tasks decreased inactivity by 52% and increased foraging time by 31% after 10 days of use. Moreover, foraging devices promoting tool use also decreased stress-related behaviors, such as abnormal and self-directed behaviors [28,32,42]. Self-directed behaviors (e.g., touching, scratching or rubbing one’s body or face), in particular, are displacement activities (i.e., species-typical behaviors exhibited out of context or in a higher frequency when animals are anxious [43]) and constitute one of the most commonly used indicators of stress or arousal in non-human primates. In fact, several studies in great apes have reported an increase in these behaviors when animals face challenging situations [44,45,46,47,48].

Foraging devices and other enrichment activities might also affect the social dynamics within the group. If only one device is available, for instance, aggression might increase in the group [28]. In contrast, when foraging devices are designed for more than one animal and/or more devices are provided, aggressive behaviors may decrease because individuals are engaged in non-agonistic activities and do not need to compete for access to the enrichment [42]. Similarly, affiliative interactions may decrease in the presence of enrichment devices if individuals spend more time manipulating the foraging device and less in social activities [42]. However, other studies have found no differences in the frequency of affiliative or aggressive behavior when providing multiple tool-based feeders to chimpanzees [32].

The study of social networks is an interesting and novel approach to measuring the effects of environmental enrichment on social dynamics [49], especially in animals with complex social lives such as non-human primates [50,51]. Nevertheless, this tool has rarely been used for this purpose, except for a few studies which investigated changes in social interactions during cognitive testing [52,53]. In particular, Whitehouse and colleagues [52] found no differences in affiliative and aggressive interactions between conditions in a group of crested macaques (*Macaca nigra*) but did detect an increase in social proximity on testing days. In contrast, Jacobson and colleagues [53] found an increase in aggressive interactions during cognitive testing, although aggression was, overall, low.

Finally, besides monitoring changes in typical behavioral patterns in the presence of enrichment devices, other indicators might be used to assess whether enrichment activities are effective at the individual level. When subjects are free to choose whether to engage in enrichment activities, for example, participation (i.e., whether subjects interact with the device or time spent interacting with it) is usually assumed to be a positive indicator of interest or motivation [24,25,54]. However, when enrichment activities are provided in a social setting, other factors might affect subject participation. For example, higher-ranking individuals or those with more dominant personality traits might have priority to access enrichment devices [29,55]. Furthermore, female chimpanzees have been reported to use tool-based enrichments more often and more efficiently than males [32,42], a pattern which has also been observed in tool-use activities in wild chimpanzees [35,56,57,58], and which they seem to share with captive bonobos [59,60].

**Figure 1:**
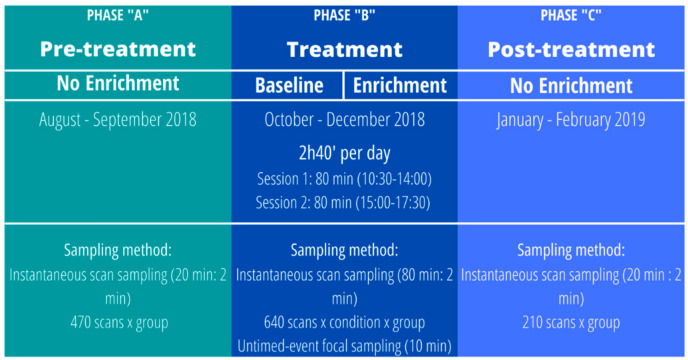
Structure of the experimental design and procedures.

The main aim of this study was to assess whether the presence and use of an artificial termite-fishing task predicted changes in individual behavioral patterns and social dynamics of sanctuary-housed chimpanzees. First, we predicted that participation in enrichment activities would vary across individuals depending on their characteristics (e.g., sex, age) and decrease through time as chimpanzee interest and motivation declined (Prediction 1, Model 1). Second, we predicted that chimpanzee solitary (Models 2–6) and social behaviors (Models 7–9) would generally differ across study phases (Figure 1). That is, if the artificial ant-fishing task had a short-term effect on chimpanzee behavior, the frequency of solitary and social behaviors should differ between the baseline and enrichment conditions during the treatment phase. Furthermore, if enrichment activities had a long-term effect on chimpanzee behavior, the frequency of behaviors in the enrichment condition (treatment phase) and after the enrichment (post-treatment phase) should differ from before the introduction (pre-treatment phase). Third, we predicted that chimpanzee solitary (Models 2b–6b) and social behaviors (Models 7b–9b) would also specifically differ depending on individual participation in enrichment activities during the treatment phase, consistently throughout the study sessions. In Models 2–9/2b–9b, in particular, we expected that the presence of the enrichment/participation would predict changes both in chimpanzee solitary behavior (i.e., increasing species-typical behaviors like tool use and feeding, decreasing undesirable behaviors like inactivity and abnormal and self-directed behaviors; Predictions 2–6/2b–6b), and in their social behavior (i.e., increasing social proximity as more individuals could simultaneously interact with the artificial termite mounds, decreasing affiliation-related behaviors as chimpanzees may spend more time interacting with the enrichment and less time in social activities and increasing aggression-related behaviors due to possible competition over the enrichment; Predictions 7–9/7b–9b). Finally, we used social network analyses to explore possible changes in chimpanzee association patterns in the presence of the enrichment (Model 10). 

## 2. Materials and Methods 

### 2.1. Subjects and Study Site

The study sample included 14 chimpanzees belonging to two mixed-sex groups of 7 individuals each (Mutamba and Bilinga groups). The Mutamba group was composed of 2 females and 5 males, with ages between 15 and 35 years (mean = 24.4 years, SD = ± 8.2 years), and the Bilinga group was composed of 3 females and 4 males, with ages between 17 and 36 years (mean = 29.1 years, SD = ± 6.7 years). All chimpanzees were housed at the Fundació Mona, a center dedicated to the rescue, rehabilitation and re-socialization of non-human primates. Supplementary Materials Table S1 contains information on demographic characteristics and background. The study chimpanzees spent their daytime hours in a 5640 m^2^ outdoor enclosure, divided into two areas (2420 m^2^ and 3220 m^2^), both covered with natural vegetation and containing wooden platforms, towers and ropes. Two observation huts (one for each enclosure) around the perimeter allowed behavioral observations of both groups. The animals also had 140 m^2^ of indoor facilities where they were housed overnight, but access was usually restricted during the daytime.

### 2.2. Experimental Procedure

We followed an ABC design (Figure 1), comparing the effects of the treatment phase (B) with a pre-test (A) and a post-test phase (C) (Models 2–9, see below). The treatment phase was structured in two alternated conditions: baseline (control days with no enrichment activity) and enrichment (days where treatment was incorporated). Eight baseline and eight enrichment days were randomly distributed for each group in a two-month period (from 22 October to 21 December 2018), excluding weekends (as visitors are more numerous than on weekdays). That is, the enrichment was available, on average, 1–2 times per week. Moreover, to avoid order effects, we also compared the effects of participation in the enrichment during the treatment phase (including baseline and enrichment days; Models 2b–9b, see below).

**Figure 2:**
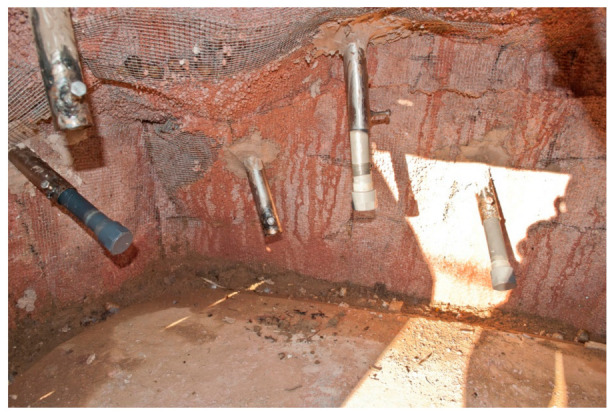
Details of the PVC tubes attached inside of the termite mound (Mutamba group). Credit: M. Llorente.

**Figure 3:**
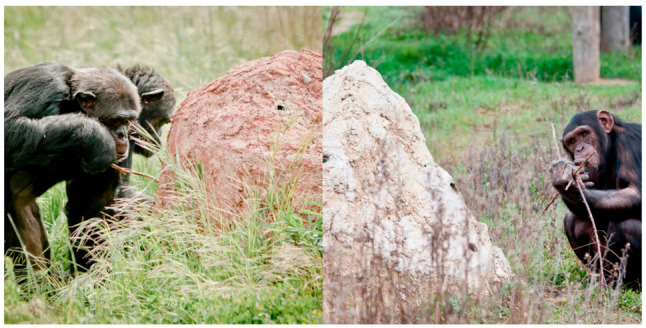
Termite mounds used during the treatment phase with the Mutamba (**right**) and Bilinga (**left**) chimpanzee groups. Credit: M. Llorente.

We used two artificial termite mounds as enrichment activity during the treatment phase. Both were made of cement and steel, with holes containing removable polyvinyl chloride tubes (length: 15–20 cm, diameter: 2.5 cm), which were attached inside the mound (Figure 2). Each group of chimpanzees had one termite mound in the enclosure. Mounds were installed approximately 10 years ago, and therefore all animals had previous experience with the task. However, they had been mostly out of use for more than 2 years before the study was conducted. That is, they remained in the enclosure, but they were seldom filled with food during this two-year period. The size of the mound and the number of holes (and therefore the tubes to be inserted) were different for each group. The mound in the Mutamba group measured approximately 2 m × 1 m width, 0.8 m height and had 9 holes, whereas the one in the Bilinga group was smaller (1 m × 1 m width and 0.8 m height) and had 5 holes (Figure 3). The tubes were filled with 10 g of honey or peanut butter mixed with 2–3 g of muesli. These quantities were removed from the total amount of food they received during the day in order to maintain a similar daily caloric intake. The use of honey or peanut butter was alternated between sessions, and both groups received the same number of sessions with each type of food. To extract the food, chimpanzees had to use sticks or branches obtained from the vegetation naturally growing in the external enclosures. No additional tools or materials were provided. The mounds were clearly visible from the observation huts around the perimeter, but the distance from the fence prevented visitors from disturbing the chimpanzees interacting with the mounds.

The termite mounds were filled in the morning, before the chimpanzees went into the outdoor enclosures, and were available throughout the daytime (from 10:30 h to 18:00 h approximately). In the baseline and enrichment days, no additional enrichment devices were provided. However, during the two-month period (i.e., non-baseline and non-enrichment days), other enrichments could be provided approximately once per week following the center’s usual routines (e.g., bottles filled with juice, fabrics with different textures, hoses filled with food). These activities have been used for a long time in the center, and they were therefore not novel for the animals. Similarly, during the pre-treatment and post-treatment phases, the termite mounds were not filled, but other enrichments could be provided as part of the center’s regular routines.

### 2.3. Behavioral Observations

#### 2.3.1. Treatment Phase: Baseline and Enrichment Conditions

On both baseline and enrichment days, we collected data for a total of 2 h 40 min per day, divided into two 80-min sessions (one in the morning between 10:30 and 14:00, and one in the afternoon between 15:00 and 17:30). We collected data in the morning because it was when chimpanzees left the indoor facilities and were first exposed to the task. However, given that the chimpanzees did not extract all the food from the termite mound right away, we also observed whether they would use the device later in the day. No observations were conducted around midday, as this was chimpanzee feeding time and usually corresponded with very low activity. 

We collected data in two ways: instantaneous scan sampling and untimed-event focal sampling [61]. First, instantaneous scan sampling with 2-minute intervals allowed us to collect data from the entire group at the same time, identifying behaviors of mid to long duration. In particular, we recorded (1) participation in the enrichment, (2) tool use, (3) feeding, (4) abnormal behaviors, (5) self-directed behaviors, (6) social proximity, (7) affiliation-related behaviors and (8) aggression-related behaviors. Details on the ethogram used for this sampling method can be found in Table 1. Some of the behaviors described above (see Table 1) were not mutually exclusive, and therefore, in each scan sampling interval, the chimpanzees could exhibit two or more behaviors at the same time. The total observation time for scan sampling was 85.33 h, equally distributed between conditions and groups, resulting in a total of 640 scans per condition and group. Secondly, we used untimed-event focal sampling [61] to collect additional data on self-directed behaviors. Specifically, we used this methodology to record rubs and scratches, which are behaviors that occur very rarely or last only a short time. Furthermore, rubs and scratches have been repeatedly associated with anxiety or arousal when chimpanzees face novel or challenging tasks [44,46,47,48], whereas the relationship between other self-directed behaviors (e.g., self-grooming) and stress is more controversial [62]. Each subject was observed for 10 minutes in the morning and 10 minutes in the afternoon. Based on the definitions and classifications provided by other authors [47,48,63], self-directed behaviors registered with untimed-event focal sampling included scratches and rubs directed towards the face and body (see detailed definition in Table 1). Following previous studies [64,65], the incidence of self-directed behaviors was quantified as the number of bouts. A bout ended when (1) the movement of the limb stopped for three or more seconds without losing contact with the body, (2) the contact between the limb and the body ceased or (3) the body target changed. Focal observations were conducted in a pseudo-randomized order to observe each chimpanzee at least once in the morning and once in the afternoon on each day.

#### 2.3.2. Pre- and Post-Treatment Phases

To further investigate the effects of termite mounds on chimpanzee well-being, we used behavioral data from before and after the introduction of the enrichment (i.e., pre- and post-treatment phases). These data were collected as part of a longitudinal study conducted at Fundació Mona [66,67], using the same ethogram and similar data collection methodology (2-min instantaneous scan sampling, 20-min sessions) that was employed during the baseline and enrichment conditions in the treatment phase. The only difference between the pre- and post-treatment phases and the treatment phase was that self-directed behaviors were only registered using the scan sampling methodology and that no focal sampling was conducted for the detection of rubs and scratches. For both pre- and post-treatment phases, we selected a similar period of time to the treatment phase (2 months) and the same number of data-collection days per group (8 days). Observations were also distributed across all days while chimpanzees were in the outdoor facilities. Nonetheless, observation time per day was variable, as observer availability was uneven during pre- and post-treatment phases. Thus, in the pre-treatment phase (August–September 2018), we were able to use a total of 470 scans per group (15.7 h of observations), whereas, in the post-treatment phase (January–February 2019), only 210 scans per group (7 h of observations) were available.

#### 2.3.3. Rank Calculation

In order to assess the subjects’ rank, we used behavioral data from a longitudinal study conducted between January 2017 and December 2019 on the same groups. In this study, instantaneous scan sampling (intervals every 2 min in 20 min sessions) was used to collect dyadic agonistic interactions with a clear winner-loser outcome, including unidirectional dominant behaviors (e.g., aggression, agonistic display, displacement) and unidirectional submissive behaviors (e.g., avoid, bared-teeth, flee). Due to observer availability, observations were not equally distributed throughout the 3-year period. In particular, total observation time for the Mutamba group was 524 h (15720 scans) and 454.57 h (13640 scans) for the Bilinga group.

#### 2.3.4. Inter-Observer Reliability

All behavioral observations were conducted by several observers, who only collected data after completing a training period and successfully passing the inter-observer reliability test (agreement between observers ≥ 85%). All data were collected using ZooMonitor [68], an application that facilitates the recording and analysis of animal behavior [69].

### 2.4. Data Analyses

Rank was calculated with the “EloRating” package [70] in R (R Core Team, Vienna, Austria, version 3.5.0), taking into account all dyadic agonistic interactions with a clear winner-loser outcome (135 interactions in the Mutamba group, and 23 in the Bilinga group). In each group, we assigned a value between 0 and 1 to every chimpanzee, with 1 corresponding to the highest-ranking subject and 0 to the lowest ranking one. Additionally, to assess rank stability, we calculated the Elo-rating repeatability score using the package “aniDom” [71]. In the Bilinga group, the repeatability score was high (r = 0.820), but given the small number of dyadic interactions in this group, and the fact that the repeatability score in the Mutamba group was very low (r = 0.279), we decided not to include rank in our models.

To investigate chimpanzee enrichment use through time, compare behavior across phases and conditions and assess the effect of participation on chimpanzee behavior, we utilized four sets of generalized linear mixed models (GLMM) [72] using the “glmmTMB” package [73] in R (R Core Team, version 3.5.0). In the first set of models, Model 1 assessed whether participation in the enrichment condition of the testing phase (i.e., the proportion of scans an individual interacted with the enrichment device in an enrichment session) varied across sessions and whether individual characteristics like sex and age predicted participation. In this model, we entered one line per individual and session (only including enrichment sessions), with session number, sex, age and time of the day (morning/afternoon) as test predictors. We further included group as a control variable and subject identity as a random effect, using a beta distribution.

In the second set of models (Models 2–9), we compared chimpanzee behaviors across phases/conditions (pre-treatment, treatment (baseline), treatment (enrichment), and post-treatment) by using phase/condition as the main predictor. In particular, these models assessed whether phase/condition predicted the occurrence of tool use (Model 2), feeding (Model 3), inactivity (Model 4), abnormal behaviors (Model 5), self-directed behaviors, (Model 6), social proximity (Model 7), affiliation-related behaviors (Model 8) and aggression-related behaviors (Model 9). In these models, we entered one line per individual and sampling scan and created binary columns (0/1) for each behavior after excluding the scans in which subjects were not visible. Therefore, the dependent variables (behaviors) were modeled with a binomial distribution. As control predictors, we entered sex, age, group, time of day (morning/afternoon) and scan number, whereas subject identity was included as a random effect.

In the third set of models (Models 2b–9b), we only used a subset of the data (i.e., baseline and enrichment conditions of the treatment phase) to assess the link between participation (i.e., the proportion of scans in each session in which an individual interacted with the enrichment device) and chimpanzee behavior. As in Models 2–9, we modeled each behavior but used participation in interaction with session number (and their main effects) as the main predictor. In particular, Models 2b–9b assessed whether participation predicted the occurrence of tool use (Model 2b), feeding (Model 3b), inactivity (Model 4b), abnormal behaviors (Model 5b), self-directed behaviors (rubs and scratches; Model 6b), social proximity (Model 7b), affiliation-related behaviors (Model 8b), aggression-related behaviors (Model 9b) and whether this effect varied across sessions. In these models, we included one line per subject and session. All the dependent variables collected with the scan sampling method (Models 2b–5b and 7b–9b) were calculated as the number of scans in which the subject performed the behavior (i.e., tool use, feeding, abnormal behaviors, social proximity, affiliation-related behaviors and aggression-related behaviors, respectively), divided by the total number of scans in which the subject was visible. Being proportions, these variables were modeled with a beta distribution. In Model 6b, instead, self-directed behaviors were collected with focal sampling, and the dependent variable was thus calculated as the total number of bouts performed in the time the subject was visible (i.e., rate of self-directed behaviors). Therefore, the dependent variable was modeled as a count (with a negative binomial distribution), adding observation time as an offset in the model. In all models (Models 2b–9b), we modeled sex, age, group and time of day (morning/afternoon) as control predictors and subject identity as a random effect. If we detected overdispersion in any of the models above (i.e., Models 2b, 5b and 9b), we transformed the predictor (participation) into a binomial predictor. If the model was still overdispersed (i.e., Models 5b and 9b), we further transformed the response into a binomial variable. We detected no overdispersion in the models presented below.

In all models, we *z*-transformed age to facilitate model convergence. To compare full models containing all predictors with null models containing only control predictors, random factors and offset terms, we used a likelihood ratio test (function “anova”) [74]. Full models were considered significantly different from null models when *p* ≤ 0.05. In the second set of models (Models 2–9), when the main categorical predictor phase had a significant effect, we used Tukey’s multiple pairwise comparisons (*p* < 0.05) to conduct post-hoc tests with the “emmeans” package [75]. In order to test our predictions, we specifically compared behaviors (i) between the baseline and the enrichment conditions of the treatment phase (i.e., short-term effect of the enrichment), (ii) between the pre-treatment phase and both the enrichment condition of the treatment phase and the post-treatment phase (i.e., long-term effect of the enrichment). In the third set of models (Models 2b–9b), if the 2-way interaction between the main predictors (participation and session number) was not significant, we removed the interaction for subsequent model iterations only, including the main effects. To rule out collinearity, we calculated variance inflation factors (VIF) [76], which were very good in all models (maximum VIF across models = 2.66).

Finally, we used social network analyses (SNA) (package “asnipe”) to assess whether the presence of the enrichment could affect the social dynamics in the groups. We focused on social proximity because we expected changes in subject associations for both participants and non-participants, depending on the presence of the enrichment. Thus, for each group and condition of the treatment phase (i.e., baseline and enrichment), we calculated the centrality degree of chimpanzees for social proximity (values between 0 and 1). Then, to assess whether the centrality degree varied in the presence of enrichment, we ran a GLMM (Model 10) using the “glmmTMB” package [73]. In this model, we entered one line per individual and per condition. As test predictors, we included the interaction of condition and group (and their main effects), adding subject identity as a random effect. We detected no converge or collinearity issues in this model (VIF = 2.26).

## 3. Results

Participation in the enrichment activity varied widely across individuals (mean = 5.65%, SD = 5.97, range = 0.00–18.25%), with 4 out of the 14 chimpanzees in our sample never being observed while interacting with the termite mounds. Supplementary Materials Tables S2 and S3 contain individual and mean values of the incidence of each behavior in the baseline and enrichment conditions of the treatment phase. Results from Models 1–10 and Models 2b–9b are presented in Supplementary Materials Tables S4–S6, respectively. Table 2 contains a summary of predictions and results for all models.

**Figure 4:**
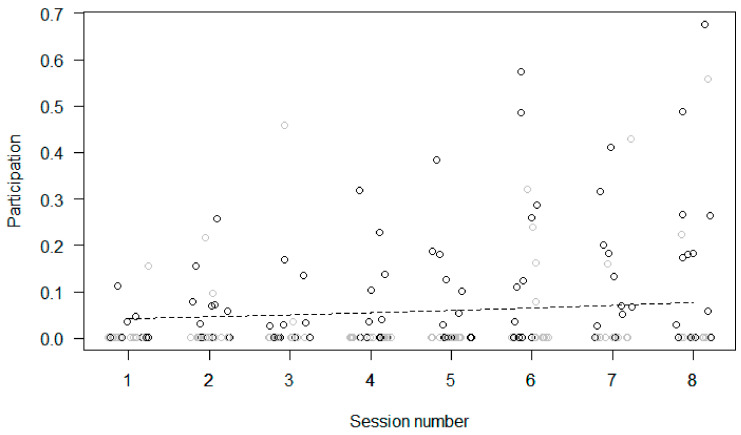
Participation in the enrichment task (i.e., proportion of scans that an individual interacted with the enrichment device in an enrichment session) as a function of time (i.e., enrichment session number) during the treatment phase. Circles represent individual responses in each enrichment session, in the morning (in black) and in the afternoon (in grey). The dashed black line represents the fitted model, which is like Model 1 but unconditional on all the other predictors that were standardized. Data were jittered horizontally to avoid overplotting.

Participation. In Model 1, the full model significantly differed from the null model. Participation in the enrichment increased across sessions (Figure 4) and was higher in the morning than in the afternoon, but sex and age had no significant effect.

Comparison of behaviors across conditions and phases (Models 2–9). In Model 2 (tool use), the full null model comparison was significant. Post-hoc comparisons indicated that tool use was more likely to occur in the presence of the enrichment (i.e., enrichment condition) than in the baseline condition. No occurrence of tool use was observed during the pre-treatment phase; thus, the frequency of this behavior was higher in the enrichment condition and post-treatment phase. Finally, tool use was more frequent in the enrichment condition than in the post-enrichment phase. In Model 3 (feeding), the full null model comparison was significant, and the post-hoc comparisons indicated that feeding was more likely to occur in the enrichment condition than in the baseline condition, pre-treatment phase and post-treatment phase. Feeding was also more likely in the post-treatment than in the pre-treatment phase. In Model 4 (inactivity), the full null model comparison was significant, and post-hoc comparisons showed that chimpanzees were more likely to be active during the enrichment condition than during the baseline condition, pre-treatment phase and post-treatment phase. Furthermore, they were also more active during the post-treatment phase than during the pre-treatment phase. In Model 5 (abnormal behaviors), the full-null model comparison was significant and post-hoc comparisons indicated that abnormal behaviors were less likely in the enrichment condition than in the baseline condition. No differences were found between the enrichment condition and either the pre- or post-enrichment phases. However, abnormal behaviors were less likely in the pre-treatment than the post-treatment phase. In Model 6 (self-directed behaviors), the full null model comparison was significant, and post-hoc comparisons revealed that self-directed behaviors were less likely in the enrichment condition than in the baseline condition. However, they also increased during the enrichment condition and the post-treatment phase, as compared to the pre-treatment phase, whereas no differences were found between the enrichment condition and the post-treatment phase. In Model 7 (social proximity), the full null model comparison was significant, and post-hoc comparisons showed no differences between the enrichment and baseline conditions. However, social proximity was more likely during the pre-treatment phase than in the enrichment condition or the post-treatment phase and more likely in the post-treatment phase than in the enrichment condition. In Model 8 (affiliation-related behaviors), the full null model comparison was significant, and post-hoc comparisons showed no differences between the enrichment and baseline conditions. Furthermore, affiliation-related behaviors were more frequent during the enrichment condition and post-treatment phase than in the pre-treatment phase, but also more frequent in the post-treatment phase than in the enrichment condition. Finally, in Model 9 (aggression-related behaviors), the full null model comparison was significant and post-hoc comparisons indicated that aggression-related behaviors did not vary between enrichment and baseline conditions nor between the enrichment condition and the post-enrichment phase. However, they increased during the enrichment condition as compared to the pre-treatment phase.

**Figure 5:**
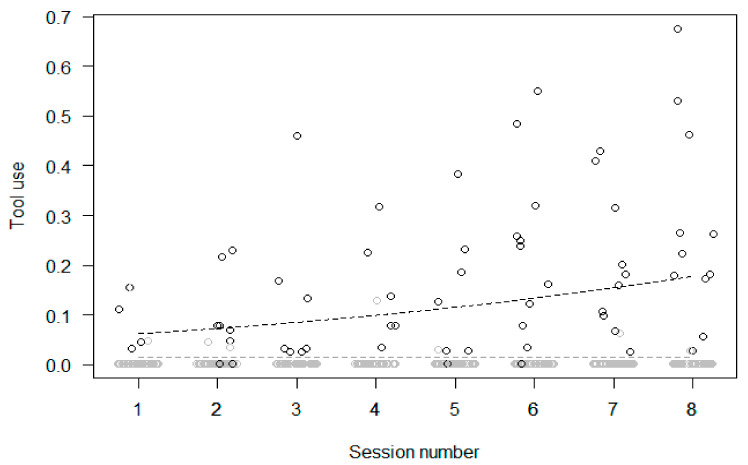
Tool use (i.e., proportion of scans where an individual engaged in tool use out of the total number of scans in which the subject was visible) as a function of time (i.e., enrichment session number) during the treatment phase. Circles represent individual responses in each enrichment session (i.e., in the morning and in the afternoon). Grey circles represent sessions in which the subjects did not participate in the enrichment task, and black circles those in which they participated. The dashed lines represent the fitted model, which is like Model 2b but unconditional on all the other predictors that were standardized. The black line represents the model for subjects participating in the enrichment task, and the grey line represents the model for those who did not participate in the task. Data were jittered horizontally to avoid overplotting.

**Figure 6:**
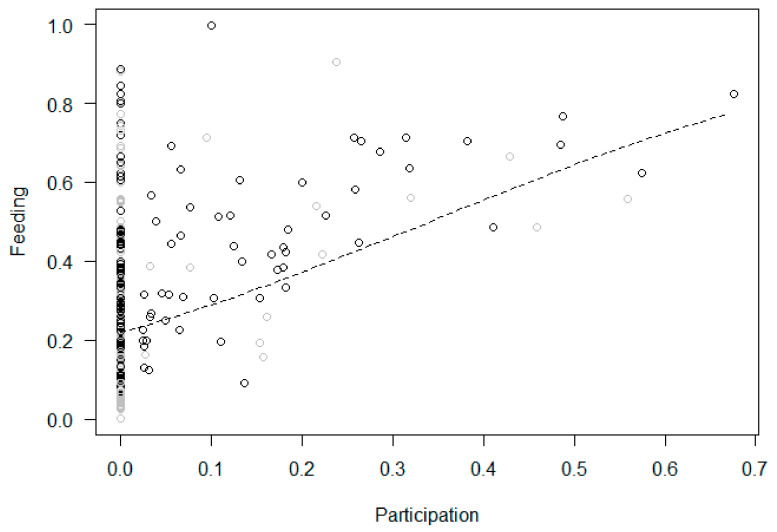
Feeding (i.e., proportion of scans that an individual was feeding, out of the total number of scans in which the subject was visible) as a function of participation (i.e., proportion of scans an individual interacted with the enrichment device in an enrichment session) during the treatment phase. Circles represent individual responses in each enrichment session in the morning (in black) and afternoon (in grey). The dashed line represents the fitted model, which is like Model 3b but unconditional on all the other predictors that were standardized.

**Figure 7:**
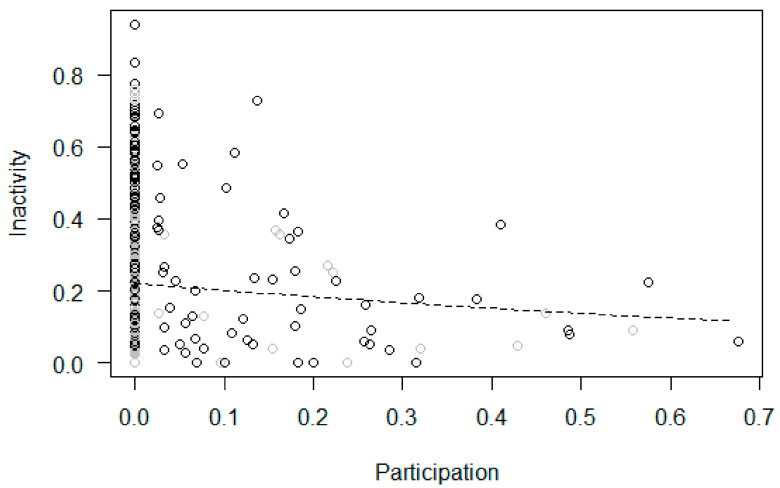
Inactivity (i.e., proportion of scans that an individual was inactive, out of the total number of scans in which the subject was visible) as a function of participation (i.e., proportion of scans an individual interacted with the enrichment device in an enrichment session) during the treatment phase. Circles represent individual responses in each enrichment session in the morning (in black) and afternoon (in grey). The dashed line represents the fitted model, which is like Model 4b but unconditional on all the other predictors that were standardized.

**Figure 8:**
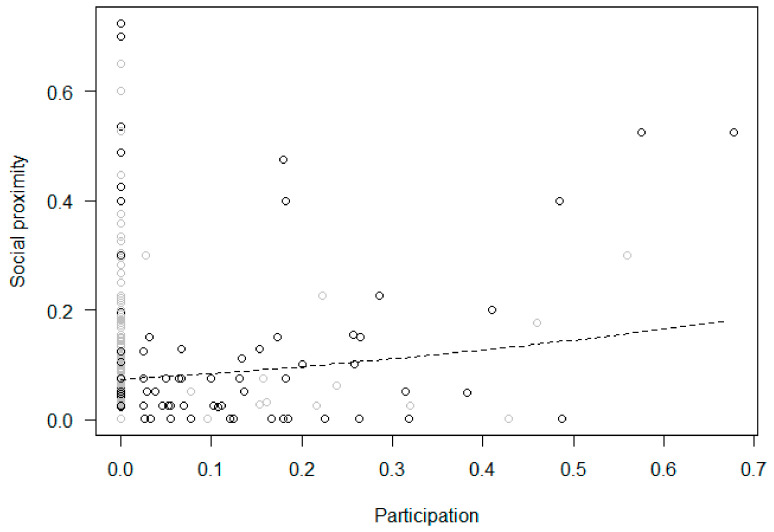
Social proximity (i.e., proportion of scans an individual was in social proximity, out of the total number of scans in which the subject was visible) as a function of participation (i.e., proportion of scans an individual interacted with the enrichment device in an enrichment session) during the treatment phase. Circles represent individual responses in each enrichment session in the morning (in black) and afternoon (in grey). The dashed line represents the fitted model, which is like Model 7b but unconditional on all the other predictors that were standardized.

Participation and changes in behaviors within the treatment phase (Models 2b–9b). In Model 2b (tool use), the full null model comparison was significant, with participation in the enrichment activities predicting a higher proportion of tool use, which increased across sessions (Figure 5). In Model 3b (feeding), the full null model comparison was significant, revealing that participation in the enrichment predicted a higher proportion of feeding, with no significant changes across sessions (Figure 6). In Model 4b (inactivity), the full null model comparison was significant, showing a link between higher participation and lower inactivity, which did not change across sessions (Figure 7). In Model 5b (abnormal behaviors), the full null model comparison was not significant. In Model 6b (self-directed behaviors (rubs and scratches)), the full model differed significantly from the null model, with the rate of self-directed behaviors decreasing across sessions but no significant effect of participation. In Model 7b (social proximity), the full null model comparison was significant, with higher participation predicting a higher proportion of proximity which did not change across sessions (Figure 8). Finally, the full null model comparison was not significant for either Model 8b (affiliation-related behaviors) or Model 9b (aggression-related behaviors).

SNA. In Model 10 (centrality degree for social proximity), the full null model comparison was not significant, revealing no significant differences in proximity patterns between conditions. Sociograms for the baseline and enrichment conditions of the treatment phase are displayed in Supplementary Materials Figure S1.

## 4. Discussion

This study assessed the impact of an artificial termite-fishing task on the behavioral patterns and social dynamics of two groups of sanctuary-housed chimpanzees. Specifically, we aimed to address: (1) whether participation in the enrichment was linked to individual differences like sex or age, and whether it would decrease across sessions (Model 1, Prediction 1), (2) if chimpanzee behavior changed across phases and conditions (pre-treatment, baseline, enrichment and post-treatment; Models 2–9, Predictions 2–9) and (3) if chimpanzee behavior changed as a function of participation during the treatment phase (Models 2b–9b, Predictions 2b–9b). Additionally, we used social network analyses to explore possible changes in chimpanzee association patterns in the presence of the enrichment (Model 10).

In contrast to our predictions, participation in the enrichment task increased across sessions and strongly varied across subjects, but with no significant effect of sex or age. As expected, the occurrence of solitary and social behaviors partly differed across phases and conditions. Specifically, tool use and feeding increased, and inactivity decreased in the presence of the enrichment, as compared to the baseline condition, the pre- and post-enrichment phases. Additional analyses revealed that changes in these behaviors were directly linked to higher participation in enrichment activities and that they were maintained across sessions. Thus, as expected, chimpanzees that used the termite mounds more often were also more likely to use tools, feed and be active, as compared to chimpanzees who did not participate in the task. We also found that participation, but not enrichment presence, was positively associated with social proximity. In contrast to our predictions, however, neither enrichment presence nor participation had a significant effect on the probability of abnormal, self-directed, affiliation-related or aggression-related behaviors. Finally, social network analyses showed that the enrichment presence did not significantly affect proximity association patterns.

As opposed to similar research conducted in captivity and in the wild, which report a higher probability of tool use activities by female chimpanzees [32,35,42,56,57,58], we found no sex differences in our termite mound experiments. However, this might depend on the small number of females in our study sample. Furthermore, although young chimpanzees in other studies spent more time in artificial termite-fishing tasks than adults [31], we did not observe age differences in task participation in this study, possibly because all the chimpanzees in our sample were adults and their age range was relatively small (i.e., 15 to 36 years). Regardless of sex or age, individual differences in enrichment use and their effects on animal behavior and welfare have been commonly reported in non-human primates [44,77]. Temperament or personality, for instance, may explain the variance in subjects’ interest towards enrichment [78,79]. For example, higher scores in trait openness have been linked to higher participation and performance in foraging puzzles, training activities and computerized activities in chimpanzees and other non-human primates [55,80,81,82,83]. Other factors that may influence subject performance include past experiences [84], rearing conditions [85] and genetics [86]. A combination of these variables could explain, for example, why four chimpanzees of our sample never interacted with the termite mounds. Therefore, future studies with larger sample sizes should include these factors when assessing the effect of enrichment on behavior and welfare. 

Previous studies show that primates easily lose interest in food-based enrichments and puzzles after relatively short exposure times [87,88,89]. In our study, however, participation did not decrease over time but actually increased across sessions. Even though animal task engagement does not necessarily provide evidence of a positive effect of the task on their welfare, it surely constitutes a basic indicator of enrichment success [25]. Therefore, the fact that participation increased across sessions indicates that chimpanzees found the termite mounds rewarding, but also that they likely required time to habituate to the task and become efficient at extracting the food. This was an unexpected outcome, considering that all chimpanzees in our sample had been exposed to this enrichment before and had the opportunity to practice the necessary skills to obtain the food from the termite mounds. One possible explanation for the increase in participation through time is that the termite mounds had not been used for a long time and were thus perceived positively as a novel stimulus, so that chimpanzee interest and efficiency would increase through sessions. Furthermore, providing the enrichment only once or twice a week (low doses), in contrast to other studies in which enrichments are continuously available (high doses), might have contributed to maintaining chimpanzee interest during the whole study period. On a side note, the chimpanzees in our study used the termite mounds more frequently in the morning than in the afternoon, likely because the food rewards in the tubes were limited, and their number decreased throughout the day.

Artificial termite-fishing tasks have already been shown to promote species-typical behaviors such as tool use and foraging in chimpanzees [28,29,31] and other great apes [90] while decreasing inactivity [29]. In our study, we observed the same increase in these behaviors when the enrichment was present (compared to the baseline condition) and in individuals who participated more in the enrichment (compared to those participating less). These results suggest a clear positive short-term effect of the enrichment on the occurrence of species-typical behaviors, especially for subjects participating in the enrichment activities. Furthermore, tool use and feeding increased, and inactivity decreased both in the post-enrichment phase and in the enrichment condition (compared to the pre-enrichment phase), suggesting a possible long-term effect of enrichment on these behaviors.

As opposed to other food-based enrichments requiring tools, we did not find a significant reduction in stress-related behaviors (abnormal or self-directed) when the enrichment was provided or if individuals participated in the enrichment [28,32,42]. Abnormal behaviors were less frequent when the enrichment was present as compared to the baseline condition but not to the pre-enrichment phase, suggesting a limited effect of the enrichment on these behaviors. In fact, they actually increased in the post-enrichment phase as compared to the pre-enrichment phase, which might suggest a negative long-term effect of enrichment activities on the occurrence of abnormal behaviors. One possible explanation for these results is that chimpanzees at Fundació Mona have a very low incidence of abnormal behaviors [67]. For example, in the baseline and enrichment conditions, the proportion of abnormal behaviors only represented less than 2.5% of their activity budget (see Supplementary Materials Table S2), which is lower than what has been reported in other captive chimpanzees (2.9 to 7.6%) [91]. It is also worth noting that a lack of change, or even an increase in abnormal and stereotypic behaviors, during or after exposure to enrichment devices is not uncommon in non-human primates [24,92,93,94]. This raises the question of whether a particular enrichment is truly beneficial if it is not directly linked to a decrease in these undesirable behaviors. In fact, some authors recommend that prior to the onset of an environmental enrichment intervention, a complete evaluation be carried out through a functional behavior assessment (FBA) [95] to identify the events contributing to the origin and maintenance of clinically relevant behavior [96,97]. Despite its potential benefits, FBA is still seldom used in applied animal welfare science with non-human primates. In addition, several authors have questioned the relationship between abnormal behavior and welfare in captive chimpanzees, arguing that the occurrence of abnormal behaviors is endemic to captive populations [91] and depends on historical variables (e.g., rearing conditions) [85]. Chimpanzees that have suffered past traumatic experiences and have spent years in impoverished environments can still engage in stereotypical or abnormal behaviors that are particularly difficult to eradicate, even years after rescue [98]. Thus, eliminating or reducing these deep-rooted behaviors may require a different approach, such as behavioral management [99,100], the application of psychological models of diagnosis and treatment [101,102] and/or pharmacological intervention [103,104].

Similar to abnormal behaviors, self-directed behaviors were less frequent when the enrichment was present than in the baseline condition, but they increased in the enrichment condition and post-enrichment phase as compared to the pre-enrichment phase, thus revealing that the presence of the enrichment had no clear effects on these behaviors. In addition, further analyses showed that higher participation was not linked to changes in the rates of rubs and scratches, which suggests that chimpanzees were not experiencing more anxiety or stress while interacting with the task. Several studies have reported an increase in self-directed behaviors when primates face novel or challenging situations [45,47,48,64]. However, all chimpanzees in our sample had previous experience with the task; despite being non-functional for two years, the termite mounds had been in the outdoor enclosures for a long time. Therefore, the component of novelty was absent in our study. Additionally, and in contrast to most studies assessing cognitive tasks in non-human primates [48,52,53,64,81], the chimpanzees were not isolated in a different location (e.g., an adjacent room) or separated from their group to perform the task. Thus, remaining in the same familiar social context likely decreased their anxiety.

Regarding the effects of enrichment on the social dynamics of the group, our results did not support our predictions. In particular, neither affiliation- nor aggression-related behaviors showed significant differences depending on enrichment presence or individual participation. These findings are consistent with those of Yamanashi and colleagues [32], who provided captive chimpanzees with tool feeders and found no differences in the frequency of affiliative or aggressive interactions when the enrichment was (or was not) available. The absence of change in the incidence of affiliation-related behaviors can be seen as a positive outcome, suggesting that the chimpanzees did not spend more time interacting with the termite mound at the expense of positive social interactions. Furthermore, the fact that several individuals could use the termite mound at the same time may explain why aggression-related behaviors did not increase, as chimpanzees did not have to compete to access the enrichment [28,42]. In fact, in line with our predictions, we did find that participation was positively associated with social proximity, confirming that the enrichment was used simultaneously by several chimpanzees and suggesting that the artificial termite mounds somehow functioned as a “gathering point” in our study groups. Unexpectedly, however, we found no differences in the frequency of social proximity between enrichment and baseline conditions. In addition, the sociograms comparing the centrality degree for social proximity in the baseline and enrichment conditions showed some variation in subject association patterns (see Figure S1), but these differences were not statistically significant. Moreover, social proximity was higher in the pre- and post-enrichment phases than in the enrichment condition. Therefore, overall, the chimpanzees spent less time in close proximity during the treatment phase, regardless of whether the enrichment was provided or not, and only those who participated more spent more time in proximity.

Recent literature claims that caregivers should aim to provide animals with opportunities to express their natural behavioral repertoire so that they experience positive welfare states [8,12,105]. In our study, although the artificial termite mounds did not significantly reduce abnormal or self-directed behaviors, they did allow chimpanzees to express species-typical behaviors such as tool use, which is rare in our groups in the absence of an enrichment that specifically promotes this behavior. Moreover, given that the behavioral effects of enrichment use were maintained across sessions, these results indicate that the artificial termite-fishing task constituted an effective enrichment, at least for the two months of the treatment period. Furthermore, the fact that participation and tool use increased across sessions suggests that, although the termite mounds were not a novel enrichment, the chimpanzees needed time to re-habituate to them and successfully retrieve food after years of not being exposed to the task. This reveals that, contrary to our expectations, the number of sessions may have been too limited to properly evaluate the effectiveness of the enrichment. By monitoring behavioral changes when using the termite mound for longer periods, it may be possible to understand how long this enrichment task may positively impact chimpanzee welfare before they lose interest, and thus optimize frequency and length of enrichment sessions as appropriate. Another limitation of our study was that because of the few agonistic interactions and low-rank stability of our chimpanzee group, we could not explore the effect of rank as we initially intended. Aggression and power conflicts were very rare in our study samples, as the chimpanzees at Fundació Mona have significant chronic social impairment caused by past traumatic experiences [66,67], and also because competition for resources is limited in small captive groups housed in large outdoor enclosures. Nevertheless, although we were unable to explore the role of rank on participation, studies on larger groups with stable hierarchies should consider this variable when studying enrichment activities in a social environment.

## 5. Conclusions

Our findings are largely in line with previous studies showing that artificial termite-fishing tasks are a successful enrichment for captive chimpanzees, promoting species-typical behaviors such as feeding and tool use and decreasing inactivity, despite not reducing abnormal or self-directed behaviors. The fact that the frequency of rubs and scratches did not increase for those individuals with higher participation suggests that the use of the termite mounds was not a source of stress. Finally, affiliation- and aggression-related behaviors were mostly not affected by the presence of the enrichment, although termite mounds appeared to work as a “gathering point”, with social proximity increasing for those who participated more often. Overall, our results show that artificial termite-fishing tasks can be an effective enrichment for sanctuary-housed chimpanzees, maintaining the subjects’ level of interest and having positive effects on their solitary behaviors without negatively affecting their social interactions.

## Figures and Tables

**Table 1:** Ethogram used during behavioral monitoring of chimpanzees.

Behavioral Category	Definition
1. Participation *	The Chimpanzee is Actively Interacting or in Contact with The Artificial Termite Mound.
2. Tool use *	To use a Mobile Element, External to the Body, to Perform a Directed Action. Includes Tool Modification and Transportation.
3. Feeding *	Searching, Locating, Handling, and Ingesting or Transporting Food. Includes Fluid Intake.
4. Inactivity	No Action or Activity, Sitting or Lying Down. Includes Self-Observation, Yawning, and Sleeping.
5. Abnormal Behaviors	Maladjusted Stereotypical Behaviors such as Rocking, Pacing, Self–Harm, Coprophagy (Eating Feces), Regurgitation, and Reingestion, Trichotillomania (Hair-Pulling), Trichotillophagia (Hair-Pulling Eating Hair), Ear-Poking, Eye-Poking.
6. Self-Directed Behaviors	**Instantaneous Scan Sampling (All Phases):** Behaviors Directed towards the Subject, such as Body Inspection, Self-Grooming, Masturbation and Scratching or Rubbing One’s Body with the Hands or Fingers. **Untimed-Event Focal Sampling (Treatment Phase):** Includes the Following Behaviors: (1) Scratches: Continuous Movement of the Hand over the Skin Involving the Ends of the Digits or Nails; (2) Rubs: Continuous Movement of the Hand over The Skin not Involving the Ends of the Digits Performed Either with the Palmar, Dorsal or Lateral Side of the Hand. This Category also Includes Self-Touches such as Nose-Wiping [48] or Face Stroking [46].
7. Social Proximity *	The Chimpanzee is at Less Than One-Arm Length from One or More Subjects while Performing any Solitary Activity, but with no Social Interaction between Subjects.
8. Affiliation-related Behaviors	Includes the Following Behaviors: (1) Grooming: Body-Cleansing Behavior (Grooming) from one Individual to Another (includes Mutual Grooming), Performed with the Upper Extremities or with the Mouth; (2) Social Play: Playful Behavior between Two or More Individuals Associated with Behavioral Indicators of Play (e.g., Play-Face, Laugh, Friendly Head Bobbing, Soft Knocking on the Ground, and Playful Chasing); (3) Sexual Behavior: Sexual Interaction, or search for Sexual Interaction, between Two Individuals Including Behaviors such as Copulation, Attempted Copulation, Genital Presentation, and Other Behaviors Directed Towards the Genitals of Another Individual; (4) Other Behaviors Identified as Affiliative, but do not fit the Criteria of Grooming, Social Play or Sexual Activity (Embrace, Feed Together and Follow).
9. Aggression-related Behaviors	Includes the Following Behaviors: (1) Agonistic Dominance: Threat-Related Behaviors such as Direct Aggression, Charging Display, Displacement and Resource Appropriation (e.g., Steal Food or Objects). Can be Accompanied by Vocalizations; (2) Agonistic Submission: Avoiding, Bared-Teeth, Displays, Food Submission (e.g., Leave/Drop Food and Move Away when Others try to Steal It), Hand-To-Mouth, Finger-To-Mouth. Can be Accompanied by Vocalizations such as Pant-Grunts. Includes Running Away from Others in Conflict Situations; (3) Other Behaviors Identified as Agonistic, but do not Fit the Criteria of Agonistic Dominance or Agonistic Submission. (Appeasing, Consolation, Reconciliation and Requesting Support).
Not Visible/Not Present	The Chimpanzee or the Behavior cannot be Identified, or the Chimpanzee is not in the Outdoor Enclosure (e.g., he is in the Sleeping Areas or in the Outdoor Cages).

* Participation, feeding and tool use were not mutually exclusive. Social proximity and all solitary behaviors (behaviors 1–6) were also not mutually exclusive (i.e., individuals could be in social proximity while simultaneously engaging in one of the solitary behaviors). Note: All behaviors were collected using 2-minute interval instantaneous scan sampling, except for self-directed behaviors, which were collected using untimed-event focal sampling.

**Table 2:** Summary of predictions and results for all models.

Models & Predictions		Supported?			
1. Participation in E…	Decreases Across Sessions is Predicted by: Sex Age Time (am/pm)	No * No No Yes			
E Presence/Participation Predicts a/an…		**E Presence (Models 2–9)**			**Participation (Models 2b–9b)**
		**Short–Term**	**Long–Term**		
		**Baseline ≠ E**	**Pre ≠ E**	**Pre ≠ Post**	
2/2b Increase in Tool Use		Yes	Yes	Yes	Yes
3/3b Increase in Feeding		Yes	Yes	Yes	Yes
4/4b Decrease in Inactivity		Yes	Yes	Yes	Yes
5/5b Decrease in Abnormal Behaviors		Yes	No	No **	No
6 Decrease in Self-Directed Behaviors		Yes	No **	No **	–
6b Decrease in Rubs and Scratches		–	–	–	No
7/7b Increase in Social Proximity		No	Yes	Yes	Yes
8/8b Decrease in Affiliation-related Behaviors		No	No **	No **	No
9/9b Increase in Aggression-related Behaviors		No	Yes	No	No
10. E Presence Predicts Changes in Proximity Social Networks		No			

* Model 1 predicted an increase in participation across sessions. ** Significant differences between phases, but in the opposite direction of the one predicted. E stands for Enrichment.

## Data Availability

The data presented in this study are available on request from the corresponding author.

## Supplementary Materials

The following are available online at https://www.mdpi.com/article/10.3390/ani11102941/s1, Figure S1: Sociograms comparing social proximity (one-arm length) across control and enriched conditions in each chimpanzee group, Table S1: Sociograms comparing social proximity (one-arm length) across control and en-riched conditions in each chimpanzee group, Tables S2 and S3: Incidence of behaviors in the base-line (S1) and enrichment (S2) conditions of the treatment phase, Table S4. Estimates, standard er-rors (SE), confidence intervals and *p*-values for Models 1–10. Reference category for phase/condition is baseline condition, for sex is female, for time is morning and for group is Bilin-ga, Table S5. Estimates, standard errors (SE) and *p*-values for post-hoc comparisons in Models 2–9. In all models, estimates are in logit-scale Table S6. Estimates, standard errors (SE), confidence in-tervals and *p*-values for Models 2b–9b. Reference category for sex is female, for time is morning and for group is Bilinga.
