# Supplementary material for: Artificial Termite-Fishing Tasks as Enrichment for Sanctuary-Housed Chimpanzees: Behavioral Effects and Impact on Welfare
Source: Animals (Basel). 2021 Oct 11;11(10):2941. doi: 10.3390/ani11102941 (PMC8532803; doi:10.3390/ani11102941)
Supplement: Supplementary file 1 [file animals-11-02941-s001.zip › Table S1.pdf]

Table S1. Biographical information on the chimpanzees from the study sample.

Subject	Sex	Group	Year of birth	Origin	Former use	Rescue year (at Mona)
Africa	F	Mutamba	1999	wild	Pet	2009
Bea	F	Bilinga	1985	wild	Entertainment	2012
Bongo	M	Mutamba	2000	captive	Entertainment	2002
Charly	M	Mutamba	1989	captive	Entertainment	2001
Cheeta	F	Bilinga	1990	wild	Entertainment	2015
Coco	F	Bilinga	1994	wild	Pet/Entertainment	2012
Juanito	M	Mutamba	2003	captive	Pet/Entertainment	2005
Marco	M	Mutamba	1984	captive	Entertainment	2001
Nico	M	Bilinga	2001	captive	Pet/Entertainment	2004
Tico	M	Bilinga	1987	wild	Entertainment	2005
Tom	M	Bilinga	1985	Wild	Entertainment	2011
Toni	M	Mutamba	1983	wild	Entertainment	2001
Victor	M	Bilinga	1982	captive	Entertainment	2006
Waty	F	Mutamba	1997	captive	Pet/Entertainment	2001
