# Supplementary material for: Artificial Termite-Fishing Tasks as Enrichment for Sanctuary-Housed Chimpanzees: Behavioral Effects and Impact on Welfare
Source: Animals (Basel). 2021 Oct 11;11(10):2941. doi: 10.3390/ani11102941 (PMC8532803; doi:10.3390/ani11102941)
Supplement: Supplementary file 1 [file animals-11-02941-s001.zip › Figure S1.pdf]

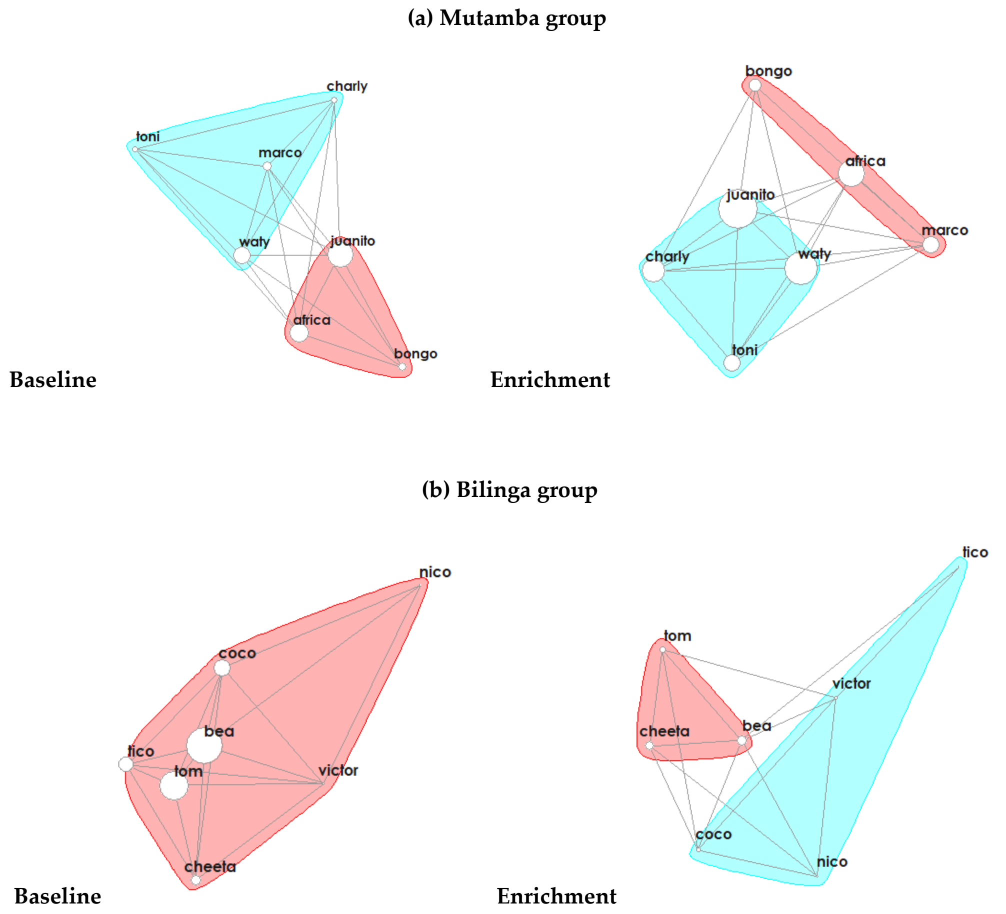

Figure S1. Sociograms comparing social proximity (one-arm length) across control and enriched conditions in each chimpanzee group. Nodes are sized according to subjects' centrality degree (strength) and edges represent dyadic associations. Colors indicate different communities in which subjects are more strongly associated (higher incidence of social proximity). N=7 in both groups.
