# Supplementary material for: Artificial Termite-Fishing Tasks as Enrichment for Sanctuary-Housed Chimpanzees: Behavioral Effects and Impact on Welfare
Source: Animals (Basel). 2021 Oct 11;11(10):2941. doi: 10.3390/ani11102941 (PMC8532803; doi:10.3390/ani11102941)
Supplement: Supplementary file 1 [file animals-11-02941-s001.zip › Tables S2 and S3.pdf]

Tables S2 and S3: Incidence of behaviors in the baseline (S1) and enrichment (S2) conditions of the treatment phase. Behaviors collected with scan sampling method (i.e., participation, tool use, feeding, inactivity, abnormal behaviors, social proximity, agonistic behaviors and affiliative behaviors) represent the percentage of scans in which the behavior was observed), whereas behaviors collected with all-occurrence focal sampling (i.e., self-directed behaviors: rubs and scratches) correspond to rates (i.e., number of self-directed behaviors/ observation time in minutes)

Table S2. Baseline condition during Phase «B» Treatment

	Scan sampling							Focal sampling
**Subject**	**Tool use**	**Feeding**	**Inactivity**	**Abnormal behaviors**	**Social proximity**	**Agonistic interactions**	**Affiliative interactions**	**Self-directed behaviors**
Africa	17.53	17.72	46.84	0.00	12.28	0.21	15.40	0.47
Bea	0.00	8.61	52.40	0.25	21.47	0.25	15.95	0.85
Bongo	0.00	18.05	39.00	0.62	4.81	2.08	8.92	0.79
Charly	8.53	26.87	17.57	0.52	2.87	0.78	23.00	0.66
Cheeta	4.01	28.90	36.42	1.93	5.89	0.00	3.28	0.74
Coco	9.05	41.29	16.95	4.30	10.29	0.00	8.35	0.98
Juanito	10.08	23.33	20.84	0.25	16.20	0.25	34.99	0.25
Marco	13.08	39.53	14.35	0.24	4.87	0.00	18.35	0.72
Nico	1.589	30.77	28.00	0.62	0.56	0.62	2.77	0.81
Tico	0.45	10.13	28.86	8.15	8.49	0.00	0.00	1.40
Tom	0.00	13.43	31.71	1.62	14.78	0.93	7.87	0.60
Toni	3.32	10.05	32.54	0.00	3.70	1.44	12.44	1.00
Victor	9.05	28.97	33.41	11.68	3.37	0.47	3.27	1.12
Waty	0.24	19.35	13.10	0.00	9.95	0.00	55.36	1.50
**Mean**	**5.49**	**22.64**	**29.43**	**2.16**	**8.54**	**0.50**	**15.00**	**0.85**

Table S3. Enrichment condition during Phase «B» Treatment

	Scan sampling								Focal sampling
**Subject**	**Participation**	**Tool use**	**Feeding**	**Inactivity**	**Abnormal behaviors**	**Social proximity**	**Agonistic interactions**	**Affiliative interactions**	**Self-directed behaviors**
Africa	18.25	0.21	29.16	35.60	0.00	14.45	0.00	13.42	0.48
Bea	0.00	0.00	9.78	44.84	0.00	6.26	0.82	12.77	0.95
Bongo	2.10	0.00	20.19	38.48	0.00	5.93	3.24	11.62	0.64
Charly	8.53	0.78	25.59	17.27	2.13	10.76	0.21	30.06	0.38
Cheeta	4.01	0.00	43.69	29.06	0.00	4.58	0.00	5.01	0.80
Coco	9.05	0.00	58.37	7.01	1.36	2.676	0.00	6.56	0.75
Juanito	10.49	0.00	38.27	8.85	0.21	18.94	1.03	37.04	0.17
Marco	13.08	0.00	45.36	10.13	0.00	8.73	0.21	15.40	0.62
Nico	1.59	0.00	36.51	20.11	0.00	1.66	1.85	2.12	1.04
Tico	0.00	0.00	20.98	16.52	6.70	0.48	0.67	0.00	1.01
Tom	0.0	0.00	17.27	20.62	0.00	3.48	1.80	6.96	0.46
Toni	0.00	0.00	4.06	28.04	0.00	8.06	0.00	28.04	0.57
Victor	10.82	0.00	44.81	18.54	2.65	2.35	1.33	2.43	0.73
Waty	1.17	0.00	15.49	15.50	0.00	16.09	0.00	53.99	0.84
**Mean**	**5.65**	**0.07**	**29.25**	**22.18**	**0.93**	**7.46**	**0.80**	**16.10**	**0.67**
