# Supplementary material for: Artificial Termite-Fishing Tasks as Enrichment for Sanctuary-Housed Chimpanzees: Behavioral Effects and Impact on Welfare
Source: Animals (Basel). 2021 Oct 11;11(10):2941. doi: 10.3390/ani11102941 (PMC8532803; doi:10.3390/ani11102941)
Supplement: Supplementary file 1 [file animals-11-02941-s001.zip › Table S4 and S5.pdf]

Table S4. Estimates, standard errors (SE), confidence intervals and *p*-values for Models 1-10. Reference category for phase/condition is baseline condition, for sex is female, for time is morning and for group is Bilinga.

**Models**	**Estimate**	**SE**	**CI (2.5%)**	**CI (97.5%)**	***p***
**Model 1: Participation** (GLMM: χ^2 = 22.54, df = 5, $p < 0.001$)					
Intercept	-3.000	0.253			
Session number	0.091	0.031	0.030	0.153	**<0.05**
Sex (male)	-0.162	0.209	-0.570	0.247	0.438
Age	-0.024	0.105	-0.230	0.183	0.823
Time (afternoon)	-0.562	0.138	-0.833	-0.291	**<0.001**
Group (Mutamba)	0.303	0.211	0.110	0.717	0.150
**Model 2: Tool use** (GLMM: $\chi 2$ = 703.42, df = 3, $p < 0.001$)					
Intercept	-9.883	0.999			
Phase Pre-treatment	-17.06	2884	-5.669	5634.744	0.995
Phase Treatment (Enrichment)	4.612	0.504	3.624	5.600	**<0.001**
Phase Post-treatment	2.491	0.636	1.244	3.738	**<0.001**
Sex (male)	0.272	0.971	-1.632	2.175	0.780
Age	-0.297	0.470	-1.218	0.625	0.528
Time (afternoon)	-1.091	0.122	-1.330	-0.851	**<0.001**
Group (Mutamba)	1.167	0.942	-0.680	3.013	0.216
Scan number	0.004	0.000	0.003	0.004	**<0.001**
**Model 3: Feeding** (GLMM: $\chi 2$ = 579.91, df = 3, $p < 0.001$)					
Intercept	-0.958	0.310			
Phase Pre-treatment	-0.796	0.055	-0.903	-0.689	**<0.001**
Phase Treatment (Enrichment)	0.418	0.044	0.332	0.504	**<0.001**
Phase Post-treatment	-0.278	0.070	-0.414	-0.141	**<0.001**
Sex (male)	0.053	0.357	-0.649	0.756	0.882
Age	-0.192	0.180	-0.544	0160	0.286
Time (afternoon)	-0.734	0.040	-0.812	-0.656	**<0.001**
Group (Mutamba)	-0.124	0.357	-0.824	0.575	0.728
Scan number	-0.000	0.000	-0.000	0.000	0.129
**Model 4: Inactivity** (GLMM: $\chi 2$ = 1609.8, df = 3, $p < 0.001$)					
Intercept	-0.254	0.241			
Phase Pre-treatment	1.206	0.043	1.121	1.290	**<0.001**
Phase Treatment (Enrichment)	-0.397	0.043	-0.482	-0.312	**<0.001**
Phase Post-treatment	-0.094	0.060	0.212	0.025	0.121
Sex (male)	-0.195	0.277	-0.738	0.347	0.480
Age	0.053	0.139	-0.220	0.325	0.704
Time (afternoon)	-0.446	0.035	-0.514	-0.378	**<0.001**
Group (Mutamba)	-0.158	0.276	-0.698	0.383	0.568
Scan number	-0.001	0.000	-0.001	-0.001	**<0.001**
**Model 5: Abnormal behaviors** (GLMM: $\chi 2$ = 53.08, df = 3, $p < 0.001$)					

Intercept	-6.253	0.749			
Phase Pre-treatment	-1.129	0.219	-1.559	-0.700	**<0.001**
Phase Treatment (Enrichment)	-0.950	0.163	-1.269	-0.631	**<0.001**
Phase Post-treatment	-0.209	0.302	-0.801	0.383	0.489
Sex (male)	1.399	0.905	-0.374	3.173	0.122
Age	0.265	0.432	-0.582	1.112	0.540
Time (afternoon)	1.380	0.153	1.080	1.679	**<0.001**
Group (Mutamba)	-2.780	0.914	-4.571	-0.989	**<0.001**
Scan number	0.002	0.000	0.001	0.003	**<0.001**
**Model 6: Self-directed behaviors** (GLMM: $\chi2$ = 266.57, df = 3, $p < 0.001$)					
Intercept	-2.585	0.426			
Phase Pre-treatment	-1.395	0.095	-1.582	-1.208	**<0.001**
Phase Treatment (Enrichment)	-0.244	0.064	-0.369	-0.119	**<0.001**
Phase Post-treatment	-0.362	0.110	-0.576	-0.147	**<0.001**
Sex (male)	0.227	0.491	-0.736	1.190	0.645
Age	-0.082	0.248	-0.568	0.405	0.743
Time (afternoon)	0.105	0.058	-0.009	0.219	0.072
Group (Mutamba)	-0.833	0.492	-1.798	0.132	0.091
Scan number	0.001	0.058	0.001	0.002	**<0.001**
**Model 7: Social proximity** (GLMM: $\chi2$ = 1127.6, df = 3, $p < 0.001$)					
Intercept	-2.770	0.330			
Phase Pre-treatment	1.610	0.063	1.486	1.73406	**<0.001**
Phase Treatment (Enrichment)	-0.154	0.070	-0.292	-0.016	**<0.05**
Phase Post-treatment	0.332	0.100	0.137	0.528	**<0.001**
Sex (male)	-0.887	0.377	-1.627	-0.148	**<0.05**
Age	-0.020	0.191	-0.395	0.354	0.915
Time (afternoon)	0.375	0.049	0.280	0.471	**<0.001**
Group (Mutamba)	0.415	0.379	-0.327	1.158	0.273
Scan number	0.001	0.000	0.001	0.001	**<0.001**
**Model 8: Affiliative interactions** (GLMM: $\chi2$ = 100.42, df = 3, $p < 0.001$)					
Intercept	-3.346	0.489			
Phase Pre-treatment	-0.474	0.074	-0.618	-0.330	**<0.001**
Phase Treatment (Enrichment)	0.071	0.057	-0.040	0.182	0.209
Phase Post-treatment	0.353	0.086	0.186	0.521	**<0.001**
Sex (male)	-1.155	0.570	-2.272	-0.038	**<0.05**
Age	-0.001	0.285	-0.559	0.557	1.000
Time (afternoon)	1.255	0.050	1.157	1.354	**<0.001**
Group (Mutamba)	1.830	0.570	0.712	2.947	**<0.05**
Scan number	0.001	0.000	0.000	0.001	**<0.001**
**Model 9: Agonistic interactions** (GLMM: $\chi2$ = 9.765, df = 3, $p < 0.05$)					
Intercept	-6.891	0.555			

Phase Pre-treatment	-0.224	0.312	-0.835	0.386	0.471
Phase Treatment (Enrichment)	0.543	0.241	0.071	1.016	**<0.05**
Phase Post-treatment	0.278	0.342	-0.392	0.949	0.416
Sex (male)	1.717	0.526	0.686	2.747	**<0.05**
Age	-0.334	0.231	-0.788	-0.079	0.149
Time (afternoon)	0.983	0.202	0.587	1.379	**<0.001**
Group (Mutamba)	-0.543	0.487	-1.500	0.412	0.265
Scan number	-0.001	0.001	-0.002	0.000	0.051
**Model 10: SNA Proximity** (GLMM: $\chi 2$ = 2.38, df = 3, p = 0.498)					
Intercept	0.492	0.112			
Condition	0.052	0.102	-0.148	0.252	0.608
Group (Mutamba)	0.068	0.159	-0.243	0.379	0.668
Condition: Group (Mutamba)	0.079	0.144	-0.204	0.362	0.582

Table S5. Estimates, standard errors (SE) and *p*-values for post-hoc comparisons in Models 2-9. In all models, estimates are in logit-scale.

**Models**	**Estimate ± SE**	***P***
**Model 2: Tool use**		
Enrichment condition > Baseline condition	-4.13 ± 0.48, -8.74 ± 0.69	**< 0.001**
Enrichment condition > Pre-treatment phase	-25.80 ± 2883.63	1.000
Pre-treatment phase < Post-treatment phase		1.000
**Model 3: Feeding**		
Enrichment condition > Baseline condition	-0.99 ± 0.17, -1.41 ± 0.17	**< 0.001**
Enrichment condition > Pre-treatment phase	-2.20 ± 0.18	**< 0.001**
Pre-treatment phase < Post-treatment phase		**< 0.001**
**Model 4: Inactivity**		
Enrichment condition < Baseline condition	-1.28 ± 0.14, -0.88 ± 0.14	**< 0.001**
Enrichment condition < Pre-treatment phase	0.32 ± 0.14	**< 0.001**
Pre-treatment phase > Post-treatment phase		**< 0.001**
**Model 5: Abnormal behaviors**		
Enrichment condition < Baseline condition	-6.69 ± 0.49, -5.74 ± 0.47	**< 0.001**
Enrichment condition = Pre-treatment phase	-6.87 ± 0.50	0.880
Pre-treatment phase < Post-treatment phase		**< 0.05**
**Model 6: Self-directed behaviors**		
Enrichment condition < Baseline condition	-2.76 ± 0.24, -2.51 ± 0.24	**< 0.001**
Enrichment condition > Pre-treatment phase	-3.91 ± 0.25	**< 0.001**
Pre-treatment phase < Post-treatment phase		**< 0.001**
**Model 7: Social proximity**		
Enrichment condition = Baseline condition	-2.74 ± 0.19, -2.59 ± 0.19	0.125
Enrichment condition < Pre-treatment phase	-0.98 ± 0.18	**< 0.001**
Pre-treatment phase > Post-treatment phase		**< 0.001**
**Model 8: Affiliative interactions**		
Enrichment condition = Baseline condition	-2.11 ± 0.28, -2.18±0.28	0.591
Enrichment condition > Pre-treatment phase	-2.65±0.28	**< 0.001**
Pre-treatment phase < Post-treatment phase		**< 0.001**
**Model 9: Agonistic interactions**		
Enrichment condition = Baseline condition	-5.60± 0.29, -6.14 ± 0.32	0.109
Enrichment condition > Pre-treatment phase	-6.37 ± 0.35	**< 0.05**
Pre-treatment phase = Post-treatment phase		0.501
